# Communities of Putative Ericoid Mycorrhizal Fungi Isolated from Alpine Dwarf Shrubs in Japan: Effects of Host Identity and Microhabitat

**DOI:** 10.1264/jsme2.ME16180

**Published:** 2017-05-20

**Authors:** Takahiko Koizumi, Kazuhide Nara

**Affiliations:** 1 Department of Natural Environmental Studies, Graduate School of Frontier Science, The University of Tokyo5–1–5 Kashiwanoha, Kashiwa, Chiba, 277–8563Japan

**Keywords:** alpine vegetation, dwarf shrub, habitat effect, host effect, mycorrhizal fungi

## Abstract

Dwarf shrubs of the family *Ericaceae* are common in arctic and alpine regions. Many of these plants are associated with ericoid mycorrhizal (ERM) fungi, which allow them to take nutrients and water from the soil under harsh environmental conditions and, thus, affect host plant survival. Despite the importance of ERM fungi to alpine plant communities, limited information is available on the effects of microhabitat and host identity on ERM fungal communities. We investigated the communities of putative ERM fungi isolated from five dwarf shrub species (*Arcterica nana*, *Diapensia lapponica*, *Empetrum nigrum*, *Loiseleuria procumbens*, and *Vaccinium vitis-idaea*) that co-occur in an alpine region of Japan, with reference to distinct microhabitats provided by large stone pine (*Pinus pumila*) shrubs (*i.e.* bare ground, the edge of stone pine shrubs, and the inside of stone pine shrubs). We obtained 703 fungal isolates from 222 individual plants. These isolates were classified into 55 operational taxonomic units (OTUs) based on the sequencing of internal transcribed spacer regions in ribosomal DNA. These putative ERM fungal communities were dominated by *Helotiales* fungi for all host species. *Cistella* and *Trimmatostroma* species, which have rarely been detected in ERM roots in previous studies, were abundant. ERM fungal communities were significantly different among microhabitats (R^2^=0.28), while the host effect explained less variance in the fungal communities after excluding the microhabitat effect (R^2^=0.17). Our results suggest that the host effect on ERM fungal communities is minor and the distributions of hosts and fungal communities may be assessed based on microhabitat conditions.

Ericoid mycorrhiza (ERM) is a symbiotic association between fungi and the roots of plants in the families *Ericaceae* and *Diapensiaceae* (*e.g. Schizocodon* and *Diapensia*) (25, 27, 43, 53, 57). The structure of ERM is characterized by hyphal coils formed in the epidermal cells of the extremely thin “hair roots” of hosts. While ERM is widespread in temperate regions, it is particularly dominant in the dwarf shrub vegetation of alpine and arctic regions (7, 16, 39, 59). Mineralization and decomposition rates in these regions are extremely low due to low temperatures, and, thus, most of the nitrogen in soil exists in organic forms that are unavailable to most plants (33). ERM host plants have the ability to utilize many forms of organic nitrogen because ERM fungi have the capacity to decompose organic matter (2, 3, 23, 34, 52). Therefore, forming a relationship with ERM fungi is essential to the success of ERM host plants, such as dwarf shrubs of the family *Ericaceae*, in alpine and arctic regions (15, 47, 59).

Various fungi have been reported as ERM mycobionts. Based on fungal isolation from ERM roots, *Helotiales* is the most dominant group (6, 14, 35, 40, 46, 50). *Rhizoscyphus ericae* (=*Hymenoscyphus ericae*) and *Oidiodendron* species have frequently been isolated from ERM roots and have been confirmed as ERM-forming mycobionts in inoculation experiments (1, 11, 24, 58). By using culture-independent methods, additional unculturable fungal groups (*e.g. Sebacinales*) have recently been identified and sequenced (1, 6, 10, 11, 15, 26, 48, 49); however, it currently remains unclear whether they form ERMs.

Research into how ERM fungal communities differ among hosts has produced inconsistent findings. For example, Bougoure *et al.* (11) demonstrated that *Calluna vulgaris* and *Vaccinium myrtillus* were associated with different ERM fungal communities in a Scots pine forest. Ishida and Nordin (29) identified such host effects on ERM fungal communities in *V. vitis-idaea* and *V. myrtillus* in northern Sweden. Toju *et al.* (56) reported significant host preferences in root-associated fungi in 3 out of 16 ericaceous plant species on Mt. Tateyama, Japan. Kjøller *et al.* (32), found no effects of host identity on the ERM fungal communities of four ericaceous hosts in northern Sweden. Walker *et al.* (59) also did not find any effects of host identity on ERM fungal communities among three ericaceous hosts in Alaska. Although we do not know the exact reason for the inconsistent findings of previous studies, the influence of microhabitats, which are quite heterogeneous in arctic and alpine regions, may account for some of the discrepancies.

Alpine habitats in Japan are often covered with sparse vegetation dominated by Japanese stone pine (*Pinus pumila*) shrubs and ericaceous dwarf shrubs. Strong winds lead to desiccation in these habitats, which makes plant establishment difficult, leaving large areas of bare ground. However, once Japanese stone pine or *Ericaceae* plants are established, their shrubs alleviate the harsh environmental conditions locally and facilitate further seedling establishment, as reported by Perkins (45). For example, Takahashi *et al.* (54) showed that the germination and establishment of Japanese stone pine seedlings were facilitated when they occurred among dwarf ericaceous shrubs, which provide ideal water conditions. Many ericaceous dwarf shrubs are found adjacent to the larger stone pine shrubs, although some ericaceous shrubs prefer other habitats. These complex and heterogeneous microhabitats in alpine ecosystems may affect belowground ERM fungal communities directly or indirectly; however, this effect has not yet been examined in detail.

In the present study, we investigated putative ERM fungal communities with reference to microhabitat and host identity using five dwarf shrub species (*Arcterica nana*, *Diapensia lapponica*, *Empetrum nigrum*, *Loiseleuria procumbens*, and *V. vitis-idaea*) co-existing in an alpine ecosystem. The hypotheses examined in this study are as follows: putative ERM fungal communities are affected more by microhabitats than by hosts, and host effects on putative ERM fungal communities become evident after excluding microhabitat effects.

## Materials and Methods

### Site description

Three study plots (0.5 ha each) were established on the exposed mountain ridges of Mt. Norikura, Kikyougahara (2,770 m a.s.l.), Daikokudake (2,770 m a.s.l.), and Fujimidake (2,790 m a.s.l.).

The mean annual temperature at our study site is −1.2°C, the lowest (−14.2°C) in January and the highest (12.0°C) in August, and mean annual precipitation is 2,738 mm, according to observations by the Japan Meteorological Agency (The Japan Meteorological Agency. 2014. Mesh Average 2010. Japan Meteorological Business Support Center: Tokyo, Japan). The site is typically covered by snow packs until late June (Norikura Observatory, pers. comm.). Five *Ericaceae* species, *i.e. A. nana*, *D. lapponica*, *E. nigrum*, *L. procumbens*, and *V. vitis-idaea*, were found in all study plots and were used in this study. ERM formation in these plant species has already been reported (25, 27, 53, 57). Larger *P. pumila* shrubs, which form ectomycorrhiza rather than ERM, were also distributed throughout these sites, providing heterogeneous microhabitats for ERM dwarf shrubs. *V. vitis-idaea* was the only species found growing under *P. pumila* shrubs. All five *Ericaceae* species of interest were found at the edges of *P. pumila* shrubs, and *A. nana*, *E. nigrum*, and *V. vitis-idaea* were dominant. *A. nana*, *D. lapponica*, *L. procumbens*, and *E. nigrum* were frequently found on bare ground away from *P. pumila* shrubs. Soil conditions on the bare ground and under pine shrubs in the three plots are summarized in Table S1.

### Sampling

In early August 2014, mature plant samples (>5×5 cm^2^) were collected from four microhabitat categories: pine shrub (the center of *P. pumila* shrubs under dense shade from pine leaves), the east edge (the eastern edge of *P. pumila* shrubs, leeward of the predominant west wind), the west edge (the western and windward edge of *P. pumila* shrubs), and open (the bare ground away from *P. pumila* shrubs). In the pine shrub habitat, we only collected *V. vitis-idaea* because no other *Ericaceae* plants were found. All five plant species were collected in the east edge, west edge, and open habitats. Individual plants were excavated with the surrounding soil, placed separately in plastic bags, and sealed and stored at 4°C during transport to the laboratory. A total of 26, 72, 76, and 48 individuals were collected in the pine shrub, east edge, west edge, and open habitats, respectively. Of the 222 plants, 60, 12, 48, 22, and 80 were *A. nana*, *D. lapponica*, *E. nigrum*, *L. procumbens*, and *V. vitis-idaea*, respectively. The number of plant samples collected for each species/microhabitat combination is listed in Table 1.

### Fungal isolation from dwarf-shrub roots

Roots were washed in running tap water and then cleaned of debris under a dissecting microscope. Two 1-cm root segments were detached from each plant and placed in a 0.2-mL tube. Root segments were sterilized with 70% ethanol for 1 min and subsequently with 30% H_2_O_2_ for 1 min (59), then washed three times in sterile distilled water. Each sterilized root segment was cut into five fragments and placed on full-strength potato dextrose agar (PDA), to which 250 ppm tetracycline was added to inhibit bacterial growth. PDA plates were maintained at room temperature under dark conditions. Each mycelium that emerged from the root fragment after a 4-month incubation was subcultured on a new PDA plate.

### DNA extraction and molecular analysis

We followed the protocol from Nara (42) for DNA extraction. Briefly, DNA was extracted from each isolated mycelium using the cetyltrimethyl ammonium bromide (CTAB) method. PCR was performed to amplify the internal transcribed spacer (ITS) regions (ITS1-5.8S-ITS2) of ribosomal DNA using the ITS5/ITS4 or ITS1F/ITS4 primer pair (20, 60), with AmpliTaq Gold (Applied Biosystems, Foster City, CA, USA). The following thermal conditions were used for PCR: an initial termination at 95°C for 2 min, followed by 40 cycles at 95°C for 30 s, at 60°C for 90 s and at 72°C for 1 min, with a final termination at 72°C for 10 min. PCR products were checked on 1.2% agarose gels under UV light. Amplified PCR products were purified, then sequenced with an ITS1 primer or ITS4 (60) using the BigDye Terminator version 3.1 kit and ABI3130 Genetic Analyzer.

### Species identification

Sequences were assembled into operational taxonomic units (OTUs) based on ≥97% similarity (51) using ATGC ver. 7 (Genetyx, Tokyo, Japan). Representative sequences (>350 bp) of individual OTUs were subjected to BLAST searches against international sequence databases (DDBJ/EMBL/GenBank) to infer their taxonomic identity. Taxonomic identity was assigned based on the BLAST results of known species in the database (≥97% similarity for the species level, ≥95% for the genus level, ≥90% for the family level, and <90% for the order or higher taxonomic level).

### Soil Analyses

We followed Miyamoto *et al.* (36) with minor modifications for soil analyses. Soil samples were air dried at room temperature, passed through a 2-mm mesh, and suspended in Milli-Q water (Millipore, Billerica, MA, USA) at a 1:10 ratio. Soil pH and electrical conductance were measured using an LAQUAtwin Compact pH meter and conductivity meter (HORIBA, Kyoto, Japan). Air-dried soils were homogenized using a zirconia ball in a 2.0-mL tube using a bead beater, then total C and total N were measured with a Flash EA 1112 CN Analyzer (AMCO, Tokyo, Japan). Summarized soil data are provided in Table S1 (soil data of edge habitats were excluded due to inadequate soil preservation after root collection).

### Statistical analyses

The occurrence of an OTU was quantified as the number of individual plants isolated from that OTU. The biased occurrence of OTUs was tested using a weighted chi-squared test, in which the number of individuals collected was used as the expected occurrence. Chao2 values, which is a species richness estimator based on the occurrence of rare taxa, were calculated for each host and microhabitat category with sample-based rarefaction analyses using Estimate S var. 8.20 (17) with 1,000 randomizations. A nonmetric multidimensional scaling (NMDS) analysis was performed based on the occurrence of OTUs to reveal the effects of habitat and host identity on ERM fungal communities. We used the Adonis function in the Vegan package of R (Oksanen, J., F.G. Blanchet, R. Kindt, P. Legendre, R.B. O’Hara, G.L. Simpson, P. Solymos, M.H.H. Stevens, and H. Wagner. 2011. vegan: community ecology package. R package version 2.0-10 Available at http://CRAN.R-project.org/package=vegan) to test for significant differences in fungal communities among habitat categories and among hosts. Singletons and doubletons were excluded from community data matrices. These statistical analyses were performed using R with 9,999 permutations, applying the Bray–Curtis distance as a community dissimilarity index.

### Data accession

The identified sequences were deposited at DDBJ under the accession numbers KY522913–KY522967.

## Results

### Fungal identity

In total, 703 isolates were obtained from 2220 root fragments. Sequences were obtained from 564 isolates, which were classified into 55 OTUs based on ≥97% ITS sequence similarity (Table 2); 12 OTUs were singletons, detected in only one plant individual. Three OTUs were assigned to the order *Capnodiales* and one to *Chaetothyriales*. All remaining fungi belonged to the order *Helotiales*, apart from two OTUs, which were assigned only at the class level. *Phialocephala fortinii* sp. 1 had the most frequent occurrence and was found in 54 plants, followed by *Cistella* sp., found in 47 (Table 2).

### ERM fungal communities in different micro-habitats

**Fig. 1:**
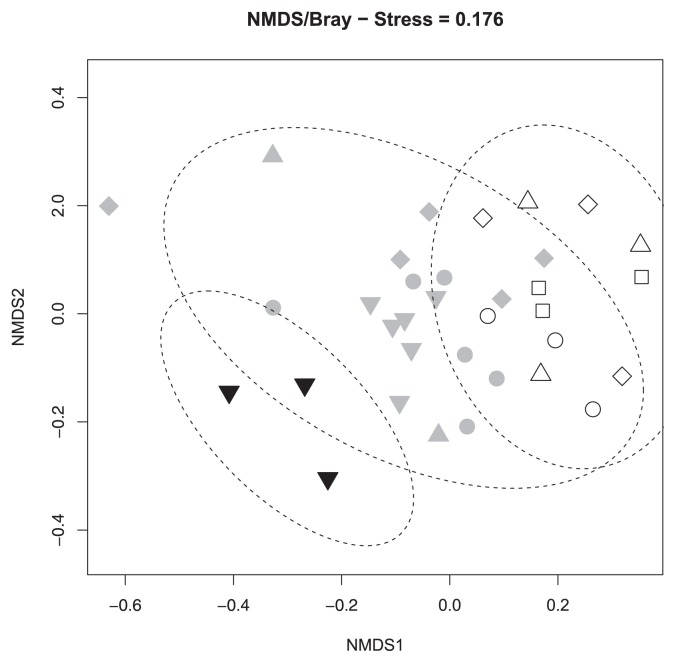
Nonmetric multidimensional scaling (NMDS) plot for ericoid mycorrhizal fungal communities in alpine dwarf shrubs. White, gray, and black colors indicate an open habitat, edge habitat, and the center of Japanese stone pine shrubs, respectively. Circles, squares, triangles, diamonds, and inverted triangles represent ERM fungal communities in *Arcterica nana*, *Diapensia lapponica*, *Empetrum nigrum*, *Loiseleuria procumbens*, and *Vaccinium vitis-idaea*, respectively. Each habitat cluster is encircled with a 95% confidence interval.

The observed OTU richness (and chao2 values) in pineshrub, east edge, west edge, and open habitats were 20 (37.3), 29 (39.9), 37 (74.4), and 16 (25.1), respectively. No significant differences were found in putative ERM fungal communities between the east edge and west edge habitats (*p*=0.326). NMDS ordination indicated that putative ERM fungal communities were clearly separated based on microhabitat (*i.e.* pine-shrub, edge, and open habitats), which was found to be significant in an Adonis test (*p*<0.001, Fig. 1). A preference for certain microhabitats was found in five OTUs (*Catenulifera* sp., *Cistella* sp., Helotiales sp. 1, *Lachnum* sp. 1, and *Trimmatostroma* sp. 1) (Table 2).

### Effects of host identity on ERM fungal communities

The observed OTU richness (and chao2 values) on *A. nana*, *D. lapponica*, *E. nigrum*, *L. procumbens*, and *V. vitis-idaea* were 27 (42.3), 4 (4.0), 21 (41.5), 9 (10.9), and 42 (67.9), respectively. Each of the hosts was uniquely associated with 6, 1, 5, 2, and 15 OTUs, respectively (Table 2). Two OTUs (*Cistella* sp. and *Phialocephala* sp. 1) were found in all hosts. Differences in occurrence between hosts were found in seven OTUs (*Hyaloscypha leuconica*, Hyaloscyphaceae sp. 2, *Lachnum* sp. 1, *Meliniomyces variabilis*, *Phialocephala fortinii* sp. 2, *Rhizoscyphus ericae* sp. 1, and *Trimmatostroma* sp. 1) (Table 2).

**Fig. 2:**
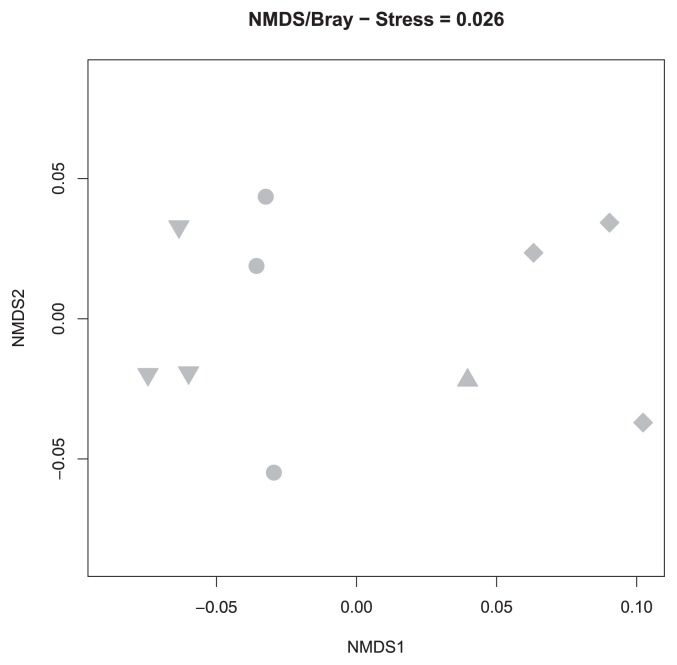
Nonmetric multidimensional scaling (NMDS) plot for ericoid mycorrhizal fungal communities in an edge habitat. Circles, triangles, diamonds, and inverted triangles represent fungal communities in *Arcterica nana*, *Empetrum nigrum*, *Loiseleuria procumbens*, and *Vaccinium vitis-idaea*, respectively.

**Fig. 3:**
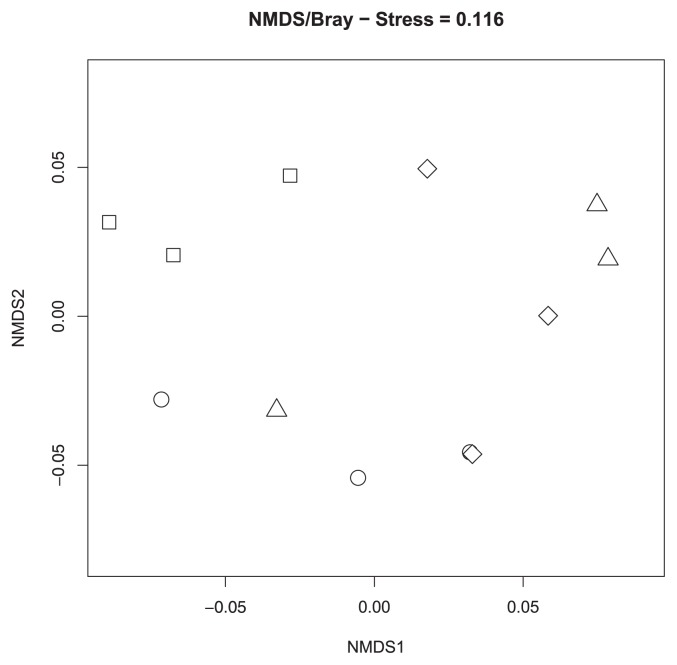
Nonmetric multidimensional scaling (NMDS) plot for ericoid mycorrhizal fungal communities in an open habitat. Circles, squares, triangles, and diamonds represent fungal communities in *Arcterica nana*, *Diapensia lapponica*, *Empetrum nigrum*, and *Loiseleuria procumbens*, respectively.

Overall, the host effect was significant in an Adonis test that did not exclude the effect of microhabitat (*p*<0.001). However, the host effect was confounded by that of microhabitat because some hosts preferred a certain microhabitat. Nevertheless, even for an Adonis test with the effects of microhabitats excluded, the host effect was significant (*p*=0.001), but explained less variance in the community data (R^2^=0.17). When only edge habitat data were used, the fungal communities of *E. nigrum* were distinct from those of *A. nana* and *V. vitis-idaea* (Fig. 2), as was further supported by an Adonis test (*p*<0.001). The fungal communities of *A. nana* and *V. vitis-idaea* were tightly clustered in the NMDS plot (Fig. 2) and were not significantly different from each other (*p*=0.800). When fungal communities were compared among hosts in just the open habitat samples, the communities in *D. lapponica* were distinct from those in other hosts (Fig. 3). A significant difference in fungal communities was detected between *D. lapponica* and all others in an Adonis test (*p*=0.014), while no significant difference was found among *A. nana*, *E. nigrum*, and *L. procumbens*.

## Discussion

Fungal communities isolated from the roots of alpine ericoid shrubs were significantly different among microhabitats (habitat: R^2^=0.28, *p*<0.001), supporting our first hypothesis that habitat has an effect on putative ERM fungal communities. Ishida and Nordin (29) found distinct fungal communities associated with *V. vitis-idaea* between pine and spruce forests. Hazard *et al.* (26) found different ERM fungal communities among sites with different land uses. Bougoure *et al.* (11) also demonstrated that ERM fungal communities varied along a vegetation gradient at the landscape scale. While these studies clearly indicate that ERM fungal communities differ by macrohabitat, the effects of microhabitat have not yet been identified. At our study sites, soil organic matter and total nitrogen were quite different among microhabitats, particularly between bare ground (open microhabitat) and under pine shrubs (Table S1). The greatest dissimilarity in putative ERM fungal communities was found between pine shrub and open habitats, while the communities in edge habitats were located between those in pine shrub and open habitats in the NMDS ordination graph (Fig. 1). Since the ability to utilize organic nutrients differs among fungal species (61), soil conditions may account for the differences observed in putative ERM fungal communities among microhabitats.

Overall, the host effect was significant in this study (R^2^=0.19, *p*<0.001), but was confounded by the effect of microhabitats because some host species showed a habitat preference. For example, *V. vitis-idaea* was the only species that was found under pine shrubs, while *D. lapponica* was mostly located in open habitats. Previous findings on potential host effects on ERM communities have been inconsistent, being significantly different in Bougoure *et al.* (11) and Ishida and Nordin (29), but not significant in Kjøller *et al.* (32) and Walker *et al.* (59). However, these previous studies did not consider the effects of microhabitats, and the contradictions among them may be due to the confounding effects of microhabitats.

In order to isolate the effects of host identity, we compared putative ERM fungal communities within each habitat category. In these analyses, host effects were not significant, except in two cases: *D. lapponica* in open habitats and *E. nigrum* in edge habitats. Although we do not know the exact reason for these exceptions, this result may partly support our second hypothesis that host effects on putative ERM fungal communities become evident after excluding microhabitat effects. In our sampling design, microhabitats were defined based on the relative location of pine shrubs due to the strong nurse–plant interaction between pine and ericaceous dwarf shrub species. However, host species may be distributed based on other factors in the field. For example, *D. lapponica* grows in exposed sites with a longer duration of the annual snowpack and is not compatible with acidic soil (37). *E. nigrum* is tolerant of snow cover, but intolerant of deep shade, and it often occurs on steep terrain where soil humidity is high (5). The habitat categories we defined here appear to contain heterogeneous geographic and edaphic conditions. Our results suggest that the host effect on ERM fungal communities is minor and the observed community differences among hosts may stem from differences in soil conditions that correlate with host distributions.

Most OTUs identified in this study were assigned to the order *Helotiales*. Nine OTUs were assigned to the *Rhizoscyphus*-*Meliniomyces* species complex, including the *Rhizoscyphus ericae* aggregate and several *Meliniomyces* species (24, 55, 58). This fungal species complex is a typical ERM fungal group (13, 24, 31, 58). Another dominant group was the *Phialocephala*-*Acephala* species complex, generally known as dark septate endophytes, which forms associations with diverse hosts (30). The dominance of these two large groups in ERM roots is congruent with previous studies (22, 59). On the other hand, we did not detect *Oidiodendron*, which is a major ERM fungal symbiont. A similar result was previously reported from Mt. Tateyama in central Japan using a culture-independent approach (56). Thus, the absence of *Oidiodendron* species may be a feature of ERM fungal communities associated with alpine dwarf shrubs in Japanese alpine regions.

Another interesting result is the dominance of *Trimmatostroma* (Capnodiales) and *Cistella* species in ERM roots. Capnodialean species have been reported to be the root-associated fungi of arctic plants, including ERM hosts, but at low frequencies (19, 59). Capnodialean species have also been reported to be leaf pathogens (12, 21) and lichen-associated fungi (12, 21, 28). *Cistella* species are typically identified as soil fungi (44) and leaf pathogens (4). Although the ERM-forming abilities of these fungal groups have not been confirmed, their dominance suggests that they play important ecological roles in association with ERM hosts.

Alpine dwarf shrubs of ericaceous plants under similar environmental conditions shared the majority of putative ERM fungi. This result indicates that most hosts are associated with generalist ERM fungi, which may, in turn, facilitate seedling establishment in a broad range of hosts and allow these hosts to co-exist. This facilitation is often reported in ectomycorrhizal systems, but not in ERM associations (8, 38, 41). This facilitation is particularly apparent in harsh environments, where mycorrhizal symbiosis is critical to plant survival (18). Generalist fungi with low host specificity are also dominant in the arctic region (9). While the ecological roles of generalist ERM fungi remain unclear, the fungal strains isolated in this study may help elucidate these roles in future research.

## Supplementary material



## Figures and Tables

**Table 1:** Plant individuals for each host and microhabitat examined

Habitat category†	Host species				

	A.n.	D.l.	E.n.	L.p.	V.v.
Pine shrub	0	0	0	0	26
East edge	23	0	16	5	28
West edge	25	0	20	5	26
Open	12	12	12	12	0

† Pine shrub, the center of *P. pumila* shrubs; East edge, the eastern edge of pine shrubs; West edge, the western edge of pine shrubs; Open, the bare ground away from pine shrubs.

Abbreviations: A.n., *Arcterica nana*; D.l., *Diapensia lapponica*; E.n., *Empetrum nigrum*; L.p., *Loiseleuria procumbens*; V.v., *Vaccinium vitis-idaea*.

**Table 2:** Species and occurrences of the fungal isolates from each habitat or host species

Taxon	Accession no.	Sequence (bp)	Best BLAST match	No. of plant individuals obtained fungal isolate								

			Accession no. (e-value, identity)	Habitat category				Host species				
	
				pine shrub	east edge	west edge	open	A.n.	D.l.	E.n.	L.p.	V.v.
Leotiomycetes sp.	KY522913	1099 bp	KP889395 (0, 89%)	1	0	1	0	1	0	0	0	1
Sordariomycetes sp.	KY522914	599 bp	KT355017 (0, 94%)	1	0	0	0	0	0	0	0	1
**Capnodiales**												
*Trimmatostroma* sp. 1	KY522915	1192 bp	KF570151 (0, 90%)	0	4	7	6**	9	0	1	2	5*
*Trimmatostroma* sp. 2	KY522916	473 bp	KF850364 (4.05E-158, 89%)	0	0	1	1	0	0	1	0	1
*Trimmatostroma* sp. 3	KY522917	822 bp	EU882733 (0, 91%)	0	0	1	0	0	0	0	0	1
**Chaetothyriales**												
*Phialophora* sp. 1	KY522918	528 bp	JQ711796 (0, 96%)	1	0	1	0	0	0	1	0	1
**Helotiales**												
*Cadophora* sp.	KY522919	823 bp	AF476977 (0, 93%)	1	2	4	0	1	0	2	0	4
*Catenulifera* sp.	KY522920	740 bp	GU727561 (0, 94%)	0	3	10	5**	4	0	9	1	4
*Cistella* sp.	KY522921	788 bp	GU174403 (0, 95%)	0	17	13	17**	12	4	13	4	14
*Cryptosporiopsis ericae*	KY522922	524 bp	JQ346985 (0, 98%)	0	3	1	1	3	0	0	0	2
*Cryptosporiopsis* sp.	KY522923	399 bp	HM030627 (0, 96%)	0	1	1	0	0	0	0	0	2
*Fontanospora eccentrica*	KY522924	771 bp	JF495222 (0, 96%)	1	2	1	0	1	0	2	0	1
Helotiaceae sp. 1	KY522925	655 bp	JN400826 (1.43E-172, 88%)	0	1	1	0	2	0	0	0	0
Helotiaceae sp. 2	KY522926	646 bp	EU292439 (0, 93%)	0	1	0	0	0	0	1	0	0
Helotiales sp. 1	KY522927	991 bp	AY822741 (0, 90%)	0	2	9	0*	3	0	0	0	8
Helotiales sp. 2	KY522928	784 bp	KC965262 (0, 91%)	0	0	3	0	2	0	0	0	1
Helotiales sp. 3	KY522929	847 bp	JX630499 (0, 89%)	2	0	1	1	1	0	0	0	3
Helotiales sp. 4	KY522930	1111 bp	FN565289 (0, 89%)	0	0	1	0	0	0	0	0	1
Helotiales sp. 5	KY522931	675 bp	JX844777 (0, 88%)	0	0	1	0	1	0	0	0	0
Helotiales sp. 6	KY522932	1121 bp	GU083254 (0, 91%)	0	1	0	0	0	0	0	0	1
*Hyaloscypha leuconica*	KY522933	514 bp	KJ649999 (0, 95%)	0	0	4	2	5	0	0	0	1*
*Hyaloscypha* sp.	KY522934	505 bp	EU292244 (0, 99%)	1	0	0	0	0	0	0	0	1
Hyaloscyphaceae sp. 1	KY522935	796 bp	KC965524 (0, 96%)	0	1	1	0	0	0	0	0	2
Hyaloscyphaceae sp. 2	KY522936	487 bp	LC035349 (0, 96%)	0	3	1	0	0	0	4	0	0*
Hyaloscyphaceae sp. 3	KY522937	533 bp	HM141054 (0, 95%)	0	1	2	0	1	0	1	0	1
Hyaloscyphaceae sp. 4	KY522938	642 bp	DQ233812 (0, 85%)	1	0	0	0	0	0	0	0	1
Hyaloscyphaceae sp. 5	KY522939	491 bp	KF617927 (3.04E-179, 90%)	1	0	0	0	0	0	0	0	1
Hyaloscyphaceae sp. 6	KY522940	742 bp	FJ827222 (0, 96%)	0	0	1	0	1	0	0	0	0
*Lachnum* sp. 1	KY522941	1110 bp	KC007291 (0, 96%)	0	3	2	6**	2	4	1	0	4**
*Lachnum* sp. 2	KY522942	519 bp	FN539070 (0, 95%)	0	0	1	0	0	0	0	0	1
*Meliniomyces variabilis*	KY522943	1120 bp	AF081435 (0, 91%)	7	5	11	0	5	0	1	1	16*
*Meliniomyces* sp. 1	KY522944	686 bp	KF617561 (0, 94%)	0	2	0	0	0	0	1	0	1
*Meliniomyces* sp. 2	KY522945	506 bp	LC131024 (0, 93%)	0	0	0	1	0	0	0	1	0
*Meliniomyces* sp. 3	KY522946	490 bp	FJ553303 (0, 97%)	0	1	0	0	1	0	0	0	0
*Microscypha ellisii*	KY522947	500 bp	KC965213 (0, 96%)	0	1	0	0	1	0	0	0	0
*Mollisia* sp. 1	KY522948	790 bp	FR773375 (0, 93%)	2	0	0	1	0	0	1	0	2
*Mollisia* sp. 2	KY522949	555 bp	AM084761 (0, 97%)	0	1	0	0	0	0	1	0	0
*Mollisia fusca*	KY522950	537 bp	AM084855 (0, 99%)	0	0	0	1	0	0	1	0	0
*Phialocephala fortinii* sp. 1	KY522951	1071 bp	AY078133 (0, 98%)	2	24	22	6	19	3	5	3	24
*Phialocephala fortinii* sp. 2	KY522952	524 bp	KX440141 (0, 97%)	0	2	5	2	7	0	0	0	2**
*Phialocephala fortinii* sp. 3	KY522953	522 bp	EU292511 (0, 99%)	0	2	2	0	1	0	0	0	3
*Phialocephala fortinii* sp. 4	KY522954	466 bp	LC131029 (0, 98%)	0	0	2	0	0	0	1	0	1
*Phialocephala sphaeroides*	KY522955	1069 bp	JQ711837 (0, 99%)	0	3	3	0	2	0	2	0	2
*Phialocephala* sp. 1	KY522956	724 bp	FR837926 (0, 98%)	0	1	0	0	0	0	0	0	1
*Phialocephala* sp. 2	KY522957	523 bp	KX611536 (0, 98%)	0	0	1	1	0	0	0	2	0
*Phialocephala* sp. 3	KY522958	791 bp	HM164649 (0, 95%)	0	0	1	0	1	0	0	0	0
*Rhizoscyphus ericae* sp. 1	KY522959	872 bp	LC131002 (0, 99%)	2	11	10	3	8	0	2	4	12*
*Rhizoscyphus ericae* sp. 2	KY522960	791 bp	KP889511 (0, 99%)	3	0	0	0	0	0	0	0	3
*Rhizoscyphus* sp. 1	KY522961	1053 bp	LC131002 (0, 94%)	1	0	3	0	1	0	0	0	3
*Rhizoscyphus* sp. 2	KY522962	542 bp	AB476467 (0, 97%)	1	1	1	0	0	0	0	1	2
*Rhizoscyphus* sp. 3	KY522963	465 bp	HQ260315 (0, 97%)	0	0	1	0	0	0	1	0	0
*Unguicularia* sp.	KY522964	696 bp	HG326612 (0, 93%)	1	0	0	0	0	0	0	0	1
Vibrisseaceae sp. 1	KY522965	748 bp	HQ260294 (0, 97%)	1	1	1	0	0	0	0	0	3
Vibrisseaceae sp. 2	KY522966	849 bp	FM207642 (0, 95%)	0	0	0	1	0	1	0	0	0
Vibrisseaceae sp. 3	KY522967	1108 bp	LC131029 (0, 94%)	1	1	0	0	1	0	0	0	1

Total †				26	72	76	48	60	12	48	22	80
Isolation ††				20	63	56	35	51	9	33	13	68
Isolation ratio (%)				76.9	87.5	73.7	72.9	85.0	75.0	68.8	59.1	85.0

* *p*<0.05,

** *p*<0.01 (chi-squared test).

Abbreviations: A.n., *Arcterica nana*; D.l., *Diapensia lapponica*; E.n., *Empetrum nigrum*; L.p., *Loiseleuria procumbens*; V.v., *Vaccinium vitis-idaea*.

† Total number of sampling points or plant individuals examined.

†† Number of sampling points or plant individuals from which fungal isolates were obtained.
