# Supplementary material for: Communities of Putative Ericoid Mycorrhizal Fungi Isolated from Alpine Dwarf Shrubs in Japan: Effects of Host Identity and Microhabitat
Source: Microbes Environ. 2017 May 20;32(2):147–53. doi: 10.1264/jsme2.ME16180 (PMC5478538; doi:10.1264/jsme2.ME16180)
Supplement: Supplementary file 1 [file 32_147_s1.pdf]

Table S1. Soil conditions of the microhabitats for three study plots

Microhabitat*	pine shrub			open		
Site	Kikyougahara	Daikokudake	Fujimidake	Kikyougahara	Daikokudake	Fujimidake
pH	3.3-3.9 (3.5)	3.4-4.1 (3.7)	3.3-3.5 (3.4)	4.4-5.1 (4.8)	4.4-5.2 (4.8)	4.3-4.9 (4.7)
Conductivity (mS/cm)	0.18-0.33 (0.28)	0.15-0.35 (0.21)	0.26-0.36 (0.31)	0.03-0.13 (0.06)	0.03-0.14 (0.07)	0.03-0.11 (0.06)
N %	0.58-1.21 (0.83)	0.58-1.82 (0.92)	0.81-1.16 (0.97)	0.04-0.56 (0.25)	0.07-0.76 (0.46)	0.05-0.52 (0.22)
C %	33.0-47.2 (42.4)	24.5-45.2 (32.0)	38.8-47.8 (44.4)	6.7-17.6 (11.1)	9.1-32.9 (14.9)	8.2-23.9 (10.7)

Abbreviations: N %, nitrogen concentration; C %, carbon concentration.

Values are minimum-maximum (mean (n=15)).

* Soil data of edge habitats are unavailable.
